# Simultaneous integrated boost of biopsy proven, MRI defined dominant intra-prostatic lesions to 95 Gray with IMRT: early results of a phase I NCI study

**DOI:** 10.1186/1748-717X-2-36

**Published:** 2007-09-18

**Authors:** Anurag K Singh, Peter Guion, Nancy Sears-Crouse, Karen Ullman, Sharon Smith, Paul S Albert, Gabor Fichtinger, Peter L Choyke, Sheng Xu, Jochen Kruecker, Bradford J Wood, Axel Krieger, Holly Ning

**Affiliations:** 1 Department of Radiation Medicine, Roswell Park Cancer Institute, Buffalo, USA; 2 Radiation Oncology Branch, National Cancer Institute, Bethesda, USA; 3 Biometric Research Branch, Division of Cancer Treatment and Diagnosis, National Cancer Institute, Bethesda, USA; 4 School of Computing, Queens University, Kingston, Canada; 5 Molecular imaging program, National Cancer Institute, National Institutes of Health, Bethesda, USA; 6 Philips Research North America, Briarcliff Manor, USA; 7 Diagnostic Radiology Dept., Clinical Center, National Institutes of Health, Bethesda, USA; 8 Department of Mechanical Engineering, Johns Hopkins University, Baltimore USA

## Abstract

**Background:**

To assess the feasibility and early toxicity of selective, IMRT-based dose escalation (simultaneous integrated boost) to biopsy proven dominant intra-prostatic lesions visible on MRI.

**Methods:**

Patients with localized prostate cancer and an abnormality within the prostate on endorectal coil MRI were eligible. All patients underwent a MRI-guided transrectal biopsy at the location of the MRI abnormality. Gold fiducial markers were also placed. Several days later patients underwent another MRI scan for fusion with the treatment planning CT scan. This fused MRI scan was used to delineate the region of the biopsy proven intra-prostatic lesion. A 3 mm expansion was performed on the intra-prostatic lesions, defined as a separate volume within the prostate. The lesion + 3 mm and the remainder of the prostate + 7 mm received 94.5/75.6 Gray (Gy) respectively in 42 fractions. Daily seed position was verified to be within 3 mm.

**Results:**

Three patients were treated. Follow-up was 18, 6, and 3 months respectively. Two patients had a single intra-prostatic lesion. One patient had 2 intra-prostatic lesions. All four intra-prostatic lesions, with margin, were successfully targeted and treated to 94.5 Gy. Two patients experienced acute RTOG grade 2 genitourinary (GU) toxicity. One had grade 1 gastrointestinal (GI) toxicity. All symptoms completely resolved by 3 months. One patient had no acute toxicity.

**Conclusion:**

These early results demonstrate the feasibility of using IMRT for simultaneous integrated boost to biopsy proven dominant intra-prostatic lesions visible on MRI. The treatment was well tolerated.

## Background

There are over 200,000 new cases and nearly 30,000 deaths each year from prostate cancer [1]. Radiation therapy (RT) is a mainstay of local therapy. It has been established that biochemical disease free survival improves with dose escalation to the prostate[2-5]. However, growing evidence indicates that normal tissue complications also increase with increasing dose[4,6,7].

The dosimetric parameters which correlate with late toxicity are being elucidated[2,7]. Advances in methods of precise radiation dose delivery, such as 3-dimensional conformal radiation therapy and intensity modulated RT (IMRT), may allow higher radiation doses to the prostate while minimizing toxicity by limiting the amount of normal tissue irradiated[8]. However, normal tissues such as the bladder and rectum abut the prostate. Therefore, dose escalation to the entire prostate results in increased doses to some portions normal tissue risking increased toxicity[2,7,9].

In principle, selective dose escalation to a dominant intra-prostatic lesion (simultaneous integrated boost) may overcome this problem of increased complications with increased dose. To date such selective dose escalation strategies have focused on the use of brachytherapy[10,11]. Several publications have discussed the theoretical aspects of simultaneous integrated boost to one or more lesions using external beam radiation therapy alone[12-14]. None of these publications, however, have reported on the results of implementing simultaneous integrated boost in patients.

This trial was undertaken to assess the feasibility and toxicity of IMRT-based simultaneous integrated boost to achieve selective intra-prostatic dose escalation to biopsy proven dominant lesions visible on endorectal coil MRI. In 42 fractions, the dominant lesion was treated to 94.5 Gy while the remainder of the prostate was treated to 75.6 Gy.

## Methods

### Eligibility and accrual

All patients underwent history and physical examination as well as routine blood work including CBC, PSA, and alkaline phosphatase. Imaging studies such as bone scan were done as warranted. Eligible patients had: 1) biopsy proven, localized adenocarcinoma of the prostate, 2) risk of lymph node metastases less than 10% by Partin tables, 3) an MRI abnormality concordant with the location of at least one sextant biopsy, and 4) were candidates for definitive external beam radiotherapy. Prior to enrollment, all patients provided written, informed consent in this IRB approved protocol.

### Study design

This is a phase I study to determine the maximum tolerated dose (MTD) with MRI-guided radiation dose escalation to regions of biopsy proven cancer within the prostate gland. There are 6 planned cohorts of 3 patients each. The dose, to the biopsy proven region of cancer evident on MRI in this first cohort, was 94.5 Gy. The dose to the region of cancer in the 6^th ^and final cohort is planned to be 152 Gy.

By design, if there are no acute dose limiting toxicities (DLT) in 3 patients then patients will be accrued to the next dose level. An acute DLT was defined as RTOG grade 3 or greater, acute GI or GU toxicity. If a DLT occurs in one of three patients then an additional 3 patients will be accrued to that dose level. If fewer than 2 of 6 patients experience an acute DLT in the expanded cohort then patients will be accrued to the next dose cohort. If 2 or more of 6 patients experience a DLT then the MTD will be exceeded and the prior, lower dose cohort will be considered the MTD.

### Magnetic resonance imaging

Endorectal coil MRI was performed at 3 Tesla using a Philips Achieva Scanner (Philips Medical Systems, Eindhoven NL.) The following pulse sequences were obtained: T2 weighted fast spin echo, MR spectroscopy, dynamic contrast enhanced MRI and delayed post contrast T1 weighted fast spin echo images. The scans were read by an experienced radiologist and determined to be positive if the T2 weighted scan was positive and one or both of the other scans were also positive at the same location.

### Biopsies and fiducial markers

All eligible patients underwent a subsequent MRI guided biopsy procedure to document the presence of prostate cancer at the location of the MRI abnormality. As previously described, biopsies were performed under direct MRI guidance [15,16] or real time ultrasound/MRI fusion[17,18]. All areas read as moderately or highly suspicious by the radiologist were biopsied. A total of 10 biopsies (half for pathology and half for our tissue bank) were allowed. Additionally, gold fiducial markers were also placed during this procedure. Generally, these markers were placed in the left middle, right middle, apex, and base of the prostate.

### Radiation therapy

Approximately one week later, with the seeds in place, patients underwent another MRI scan which was fused with the treatment planning CT scan. MR and CT fusion was done using the Eclipse treatment planning software and manually verified and optimized by checking the seed position in both scans. This fused MRI scan was used to delineate the region of the biopsy proven intra-prostatic lesion. A 3 mm expansion was performed on this intra-prostatic lesion, defined as a separate volume within the prostate. The lesion + 3 mm received 94.5 Gy in 2.25 Gy daily fractions while the remainder of the prostate + 7 mm received 75.6 Gy in 1.8 Gy daily fractions. If needed, the seminal vesicles were allowed to be treated to 54 Gy.

No volume 4 mm beyond the lesion + 3 mm was allowed to receive a dose beyond 75.6 Gy. Less than 25% of the rectal volume was allowed to receive more than 70 Gy. No more than 40% of the bladder was allowed to receive more than 65 Gy. Maximum point dose to the rectum and bladder was limited to 80.5 Gy. Attempts were made to limit the prostatic urethra to 80 Gy. Though this did not occur in the current cohort, if the urethral constraint was not met, then specific authorization would be required by the principal investigator to proceed with treatment.

Prior to each fraction, seed position was verified to be within 3 mm of the planned position by electronic portal imaging.

### On treatment and follow-up evaluations

Patients were seen by a physician weekly while on treatment. Upon completion of therapy, follow-up visits occurred at 2, 4, and 8 weeks, 3 months, 6 months, then every 6 months until 3 years. Formal toxicity measures were obtained and recorded at baseline, at weeks 5 and 7 of therapy (when radiation therapy was nearly complete), and at each follow-up visit. These toxicity measures included Radiation Therapy Oncology Group (RTOG) acute (within 120 days of completion of radiation) and late toxicity grading and Expanded Prostate Cancer Index Composite (EPIC) self-assessment questionnaires[19].

### Statistical analysis

Summary statistics, such as sample proportions, listing of values for each patient, and range of values were used to describe the patient characteristics. Characteristics of radiation dosimetry were described using maximum dose and percent volume of structures receiving greater than threshold dose.

## Results

**Figure 1:**
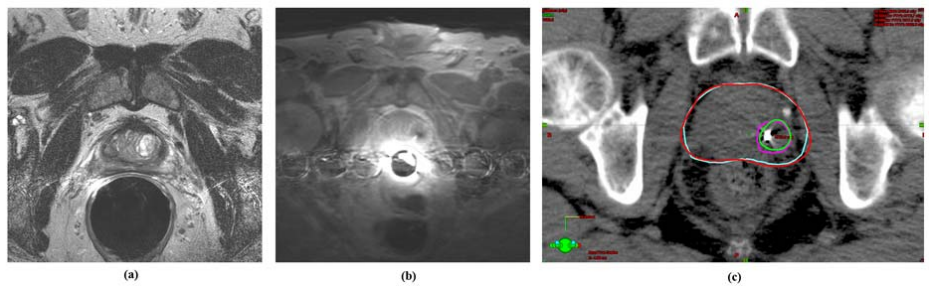
**1a: **Dynamic Contrast Enhanced (DCE) MR image showing region of increased gadolinium uptake in the left peripheral zone. **1b: **MRI guided biopsy showing needle in the same region as in frame a. Pathology showed Gleason Score 7 disease. Immediately afterward, a fiducial marker was also placed in this location. **1c: **Treatment planning image showing a fiducial marker in same region as figure a and b. The target was defined by fusing a treatment planning MRI (not shown and without an endorectal coil in place) with the treatment planning CT. The isodose lines are shown on the CT where the fiducial marker is best seen. The planning target volume of the intra-prostatic lesion is shown in fuschia. The 94.5 Gy isodose line is shown in green. The planning target volume of the prostate is shown in blue. The 75.6 Gy isodose line is shown in red.

**Figure 2:**
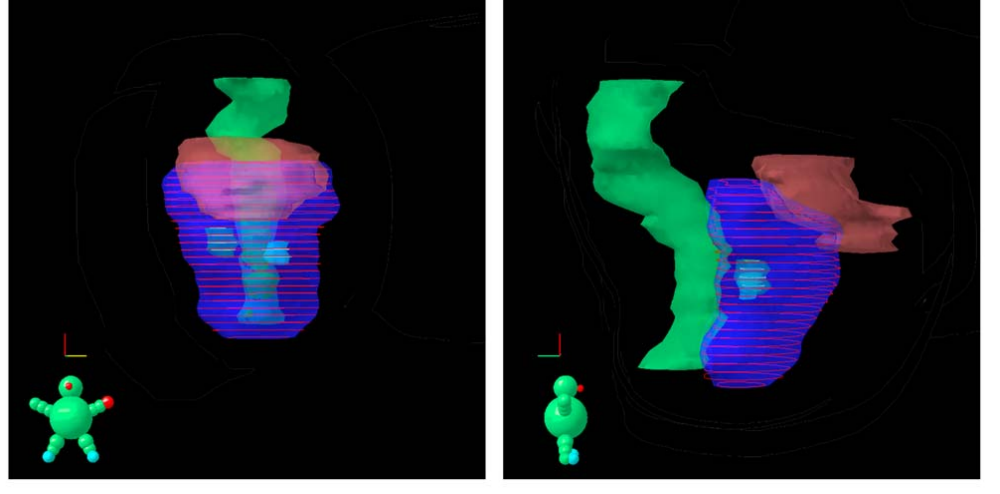
Radiation dose plan showing anterior view on the left panel and lateral view on the right panel. This 56 year old patient had 2 areas on prostate MRI suspicious for cancer. MR guided biopsies of these suspicious areas were performed. Both suspicious areas were positive for Gleason Score 6 prostate cancer. The 94.5 Gy dose clouds of the simultaneous integrated boost are seen in the left and right mid gland as yellow rings around the contoured MR abnormalities of biopsy proven cancer. The 75.6 Gy dose cloud covering the remainder of the prostate is represented by red rings. The rectum (green) and bladder (light brown) are also shown.

Three patients were treated. Follow-up was 18, 6, and 3 months respectively. The first and third patients had a single, biopsy confirmed intra-prostatic lesion. The second patient had 2 intra-prostatic lesions. All 4 intra-prostatic lesions, with margin, were successfully targeted by MR guided biopsy, (Figures 1a–1c.) These intra-prostate lesions were targeted, biopsied, marked with a fiducial marker, and treated to 94.5 Gy while the remainder of the whole prostate was treated to a minimum of 75.6 Gy (Figure 2.) Maximum and minimum doses to critical structures are summarized in Table 1. Of the planning target volumes, at least 97% of prostate and 90% lesion volumes were covered by the prescription dose.

**Table 1:** Doses to Critical Structures

	**Rectum**		**Bladder**		**Uretha**
**Patient**	**Maximum Dose (Gy)**	**% Vol > 70 Gy**	**Maximum Dose (Gy)**	**% Vol > 65 Gy (cubic centimeters (cc) > 65 Gy)**	**Maximum Dose (Gy)**

**1**	80.4	7.34%	80.3	10.42% (20.13 cc)	78.4
**2**	78.1	9.4%	78.2	40% (18.4 cc)	78.5
**3**	79.9	6.18%	80.5	10.67% (21.56 cc)	79.2

Two patients experienced acute RTOG grade 2 GU toxicity. One had grade 1 GI toxicity. These symptoms completely resolved by 3 months. One patient had no acute toxicity.

**Figure 3:**
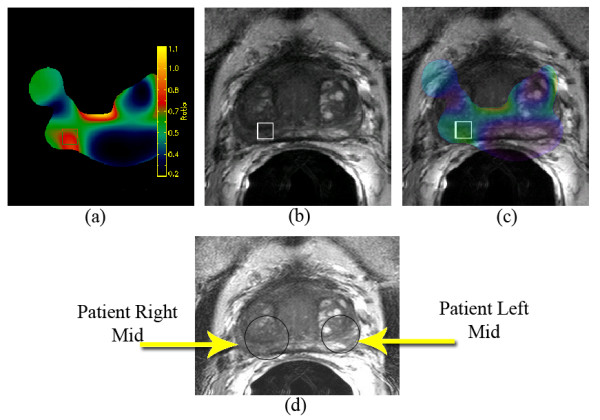
False positive endorectal coil MRI lesion with contralateral malignant lesion. **3a**: Color chart obtained from the MR Spectroscopy showing an elevated ratio of choline to citrate (depicted in red) in the right mid gland of the prostate. **3b: **Area of the right mid gland (shown as a square in both a and b) on the T2 weighted MRI of the prostate where a high choline to citrate ratio was observed, indicating a highly suspicious for prostate cancer region of low signal intensity. **3c: **Overlay of the T2 MRI and the color map of the MR Spectroscopy. **3d: **T2 MRI of the prostate. To avoid any suspicion of sampling error, 8 biopsies were performed on the left and right mid glands. All 4 biopsies from the left mid (read as moderately suspicious for prostate cancer) were positive for Gleason Score 6 disease. All 4 biopsies from the right mid gland (read as highly suspicious for prostate cancer) demonstrated only chronic inflammation.

In patient 1, the single targeted biopsy was positive. In patient 2, both highly suspicious lesions were positive (Figure 2.) In patient 3, the highly suspicious lesion on MR yielded no malignant tissue histologically (but did demonstrate chronic inflammation) on 4 targeted biopsies while the moderately suspicious lesion on MR yielded 4 of 4 biopsies positive for Gleason score 6 prostate cancer (Figure 3.)

## Discussion

The early results of this trial demonstrate the feasibility of using IMRT-based simultaneous integrated boost to selectively increase dose to biopsy proven dominant intra-prostatic lesions visible on MRI. The treatment was well tolerated. All patients achieved resolution of treatment related gastrointestinal and genitourinary symptoms on the RTOG scale.

These findings are consistent with previous dosimetric analyses which reported that, in theory, a external beam radiation therapy based simultaneous integrated boost dose to a MRI defined dominant intra-prostatic lesion(s) should have acceptable toxicity[12-14].

Pickett et al. showed that an early form of IMRT could be used to deliver 90 Gy to a single MRI-defined intra-prostatic lesion while treating the rest of the prostate to 70 Gy in 1.8 Gy daily fractions. In fact, this plan with simultaneous integrated boost to the dominant intra-prostatic lesion actually produced a slightly lower rectal dose than a standard three dimensional conformal radiation plan giving only 70 Gy to the prostate[13]. Recently, van Lin et al. performed a similar analysis on 5 patient data sets comparing IMRT plans which gave 78 Gy to the prostate with IMRT plans giving 70 Gy to the whole prostate while giving a 90 Gy simultaneous integrated boost to a single MR-defined dominant intra-prostatic lesion in each patient. Echoing Pickett et al., the authors found that rectal doses, and therefore presumably complications, would have been lower in the group receiving simultaneous integrated boost[12].

In the current study, 2 patients had a single intra-prostatic lesion. One patient had 2 MRI defined, biopsy proven intra-prostatic lesions. The successful treatment of this patient demonstrates the practical ability to safely deliver simultaneous integrated boosts to 2 intraprostatic lesions without significant toxicity. The theoretical feasibility of this approach was reported by Xia et al. who ran multiple IMRT plans on a single selected case with 2 intraprostatic lesions. The authors concluded that it was technically feasible to concurrently treat multiple selected high-risk regions within the prostate to 90 Gy and the remaining prostate to 75.6 Gy. Doses to the rectum and the bladder suggested that Grade 2 complications should occur in significantly less than 10%[14].

Consistent with these theoretical findings, all 3 patients in the current study achieved resolution of acute treatment related gastrointestinal and genitourinary symptoms as described by the RTOG scale. No late toxicities have been observed. In fact, in follow up, one patient has shown marked improvement from baseline, pre-treatment symptoms of urinary frequency.

Certainly, these early results are encouraging. However, 2 substantial hurdles remain prior to wide implementation of this approach. First, it remains unclear how well MR scans differentiate regions of prostate cancer from regions of prostate inflammation. Anastasiadis et al., in a series of prostate biopsies performed under direct MR guidance, noted that prostatitis and prostate cancer have a quite similar appearance on MR[20].

Our data concur with this finding[21]. Figure 3 illustrates that MR spectroscopy and DCE imaging are often unable to discriminate cancer from inflammation of the prostate. Therefore, intra-prostatic lesions as defined by MR should not be targeted for simultaneous integrated boost in the absence of biopsy proven cancer in that region. Second, the long term effects of this treatment strategy, though assumed to be minimal, have yet to be established.

Simultaneous integrated prostate boost with IMRT therefore remains experimental and should only be performed on IRB approved, prospective trials with appropriate informed consent and planned long term follow-up.

## Conclusion

These early results demonstrate the feasibility, with excellent early toxicity, of using IMRT for simultaneous integrated boost to biopsy proven prostate cancer visible on MRI. Long term follow up with larger numbers of patients are needed prior to wide implementation of this technique. Simultaneous integrated IMRT boost to intra-prostatic lesions should only be undertaken on institutional review board approved trials with image guided biopsy evidence of disease in that location.

## Competing interests

The author(s) declare that they have no competing interests.
